# MGluR5 Mediates the Interaction between Late-LTP, Network Activity, and Learning

**DOI:** 10.1371/journal.pone.0002155

**Published:** 2008-05-14

**Authors:** Arthur Bikbaev, Sergey Neyman, Richard Teke Ngomba, Jeffrey Conn, Ferdinando Nicoletti, Denise Manahan-Vaughan

**Affiliations:** 1 Department of Experimental Neurophysiology, Medical Faculty, Ruhr University Bochum, Bochum, Germany; 2 International Graduate School of Neuroscience, Ruhr University Bochum, Bochum, Germany; 3 Synaptic Plasticity Research Group, Institute for Physiology (Charité), Humboldt University, Berlin, Germany; 4 Instituto Neurologico Mediterraneo (INM), Neuromed, Pozzilli, Italy; 5 Department of Pharmacology, Vanderbilt University Medical Center, Nashville, Tennessee, United States of America; 6 Department of Human Physiology and Pharmacology, University of Rome “La Sapienza,” Rome, Italy; Medical College of Georgia, United States of America

## Abstract

Hippocampal synaptic plasticity and learning are strongly regulated by metabotropic glutamate receptors (mGluRs) and particularly by mGluR5. Here, we investigated the mechanisms underlying mGluR5-modulation of these phenomena. Prolonged pharmacological blockade of mGluR5 with MPEP produced a profound impairment of spatial memory. Effects were associated with 1) a reduction of mGluR1a-expression in the dentate gyrus; 2) impaired dentate gyrus LTP; 3) enhanced CA1-LTP and 4) suppressed theta (5–10 Hz) and gamma (30–100 Hz) oscillations in the dentate gyrus. Allosteric potentiation of mGluR1 after mGluR5 blockade significantly ameliorated dentate gyrus LTP, as well as suppression of gamma oscillatory activity. CA3-lesioning prevented MPEP effects on CA1-LTP, suggesting that plasticity levels in CA1 are driven by mGluR5-dependent synaptic and network activity in the dentate gyrus. These data support the hypothesis that prolonged mGluR5-inactivation causes altered hippocampal LTP levels and network activity, which is mediated in part by impaired mGluR1-expression in the dentate gyrus. The consequence is impairment of long-term learning.

## Introduction

Hippocampus-based learning and memory is likely to be encoded by two forms of hippocampal synaptic plasticity: long-term potentiation (LTP) and long-term depression (LTD) [1]–[2]. N-methyl D-aspartate receptor (NMDAR)-dependent forms of LTP and LTD are induced by patterned electrical stimulation of perforant path or Schaffer collateral/commissural fibres and endure for days and weeks *in vivo* [3]–[5]. Although the role of the metabotropic glutamate receptors (mGluRs) in hippocampal synaptic plasticity has proved a point of controversy in *in vitro* studies, considerable consistency in support of a critical role for these receptors in the persistence of synaptic plasticity *in vivo* is evident [6]–[11].

As members of family C of the G-protein coupled receptors, group I mGluRs possess a large extracellular domain containing an orthosteric binding site for glutamate, a heptahelical transmembrane domain that contains an allosteric modulatory binding site, and an intracellular C-terminus that interacts with anchoring/scaffolding proteins and controls the constitutive activity of the mGluR [12]–[13]. Group I mGluRs, comprising mGluR1 and mGluR5, are located primarily postsynaptically and coupled preferentially to G_q/11_ and its effectors, such as phospholipase C. Activation of group I mGluRs increases intracellular Ca^2+^ concentration via two distinct mechanisms: potentiation of NMDAR currents and Ca^2+^ release from intracellular pools (see for review: [12]–[14]).

In as much as elevation in intracellular calcium levels determines the expression of NMDAR-dependent hippocampal LTP and LTD [15], both of which are protein synthesis dependent [16]–[17], changes in cytosolic calcium concentration may be intrinsically involved in the cellular mechanisms underlying information storage in the mammalian brain. The impairments of both LTP and spatial learning through mGluR5 antagonism [11], [18] may also be related to alterations in the surface expression or cycling of these receptors [19]. Group I mGluRs play an important role in the regulation of network activity in the hippocampus [20]–[22]. Functional disruptions of these receptors may alter intrinsic hippocampal network activity that in turn affects the ability of the hippocampus to engage in information storage.

We set about to address these possibilities using *in vitro* recordings from the CA1 hippocampal slice preparation and chronic electrophysiological recordings from two sub-regions of the hippocampus of the adult rat. Studies were conducted in parallel with analysis of learning in the 8-arm radial maze and with biochemical analysis. Consequences of mGluR5 inactivation for hippocampal network activity were assessed using analysis of intrahippocampal theta and gamma oscillations.

Our data reveal that regulation by mGluR5 of hippocampal synaptic plasticity occurs both at the NMDA receptor-dependent phase and at the protein synthesis-dependent phase of LTP. The decline in both short-term and long-term memory, which is observed following pharmacological blockade of mGluR5, is coupled with *deficits* in late-LTP in the dentate gyrus and an *enhancement* of LTP in the CA1 region. This effect is in turn associated with an inhibition of mGluR1a receptor expression and alterations in theta-gamma activity in the dentate gyrus. We postulate that the down-regulation of mGluR1a is a key factor in the effects mediated by prolonged mGluR5 blockade: treatment with an mGluR1a potentiator reversed effects in the dentate gyrus, and CA3-lesioning prevented effects in the CA1 region. Our data provide a strong link between theta-gamma activity, LTP expression, and the encoding of short and long-term memory in the hippocampus, and support that mGluR5 strongly regulates these phenomena by a mechanism involving control of the expression of mGluR1.

## Results

### Prolonged mGluR5 antagonism inhibits working and reference memory performance

**Figure 1:**
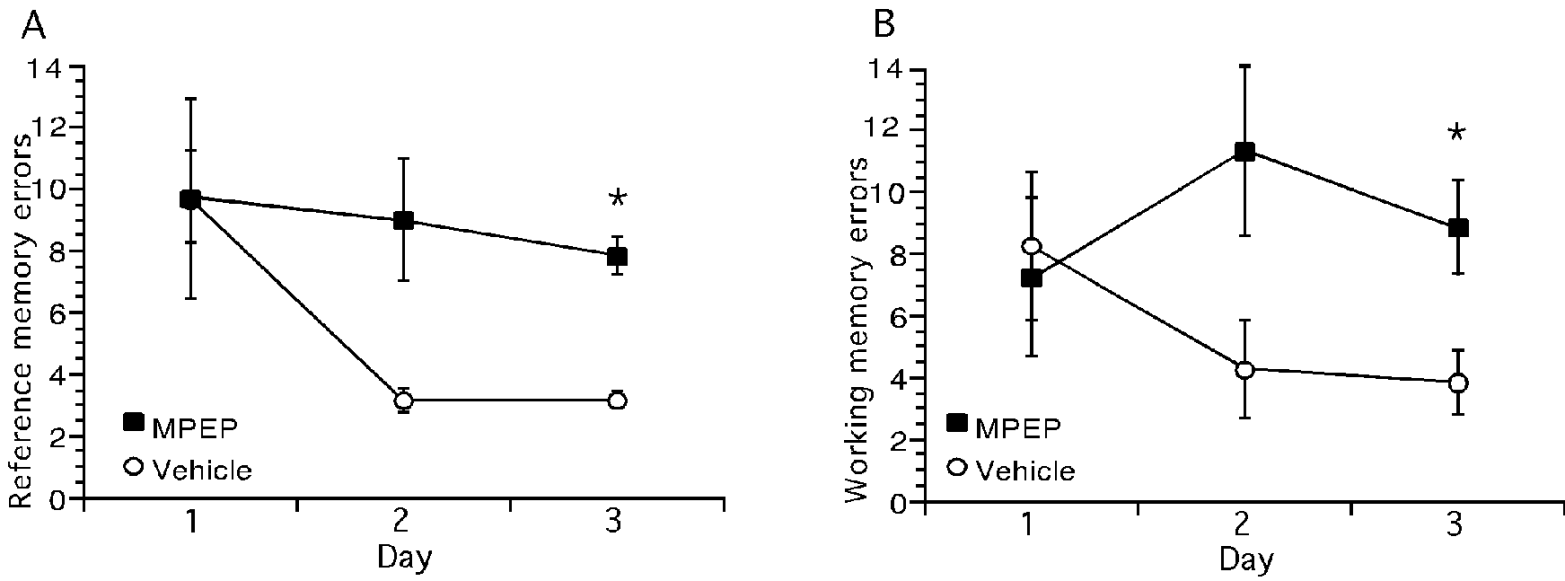
Prolonged mGluR5 antagonism *in vivo* inhibits working and reference memory performance. A, B. MPEP was given daily (1.8 µg, i.c.v.), 30 min prior to testing of learning performance in an 8-arm radial maze where only 4 arms were baited with food. By the third trial day a significant impairment of both reference (A) and working memory (B) was apparent. Asterices denote statistical significance (p<0.05). Data are represented as mean±S.E.M.

Daily application of 2-methyl-6-(phenylethynyl)pyridine (MPEP, 1.8 µg, i.c.v), the non-competitive mGluR5 antagonist [23], has been shown previously to cause impairments of memory performance in the 8-arm radial maze [11], [18]. Effects first become apparent by the third day of MPEP treatment and become more pronounced when treatment is continued over several days. Our objective was to examine changes in hippocampal function that become apparent in parallel with spatial learning deficits. Therefore, to confirm that comparative learning deficits were also seen in the animals used in the present study, we followed learning performance in the radial maze for three days during which the animals received either MPEP (1.8 µg, i.c.v, n = 9) or vehicle (n = 7) as a daily injection 30 minutes before training in the maze occurred (Figure 1). Consistent with our previous observations, impairment of reference memory performance became apparent by the third day of treatment (t-test: p<0.001, ANOVA of 3 days: F_1,2_ = 4.81, p<0.05). Working memory was also significantly impaired by day 3 (t-test: p<0.05, ANOVA of 3 days: F_1,2_ = 6.51, p<0.05). The effect on reference memory was not accompanied by any changes in locomotion, rearing, grooming, or defecation (data not shown), in agreement with our previous observations [11], which suggests that the concentration of MPEP used inhibits learning without causing anxiolysis or eliciting changes in motor function.

### Prolonged antagonism of mGluR5 leads to inhibition of mGluR1a expression in the dentate gyrus; expression of mGluR5, mGluR2/3 and NR2A remains unchanged in the CA1 region

We first investigated the molecular basis for the impairment of spatial memory seen following prolonged mGluR5 antagonism. Induction of LTP *in vivo* was reported previously to lead to enhanced expression of mGluR5 and reduced expression of mGluR2/3 in the dentate gyrus and CA1 region [19]. One possibility is that mGluR5 antagonism leads to an alteration in mGluR5 or other glutamate receptor protein expression. We therefore examined the expression of NR2A, mGluR5, mGluR1a and mGluR2/3 receptor proteins in the hippocampus (CA1 and dentate gyrus) of rats treated with MPEP or vehicle once daily for 3 days (both n = 6). Brains were removed 24h after the final injection. This protocol was chosen because learning is impaired after 3 daily injections of MPEP (Figure 1).

**Figure 2:**
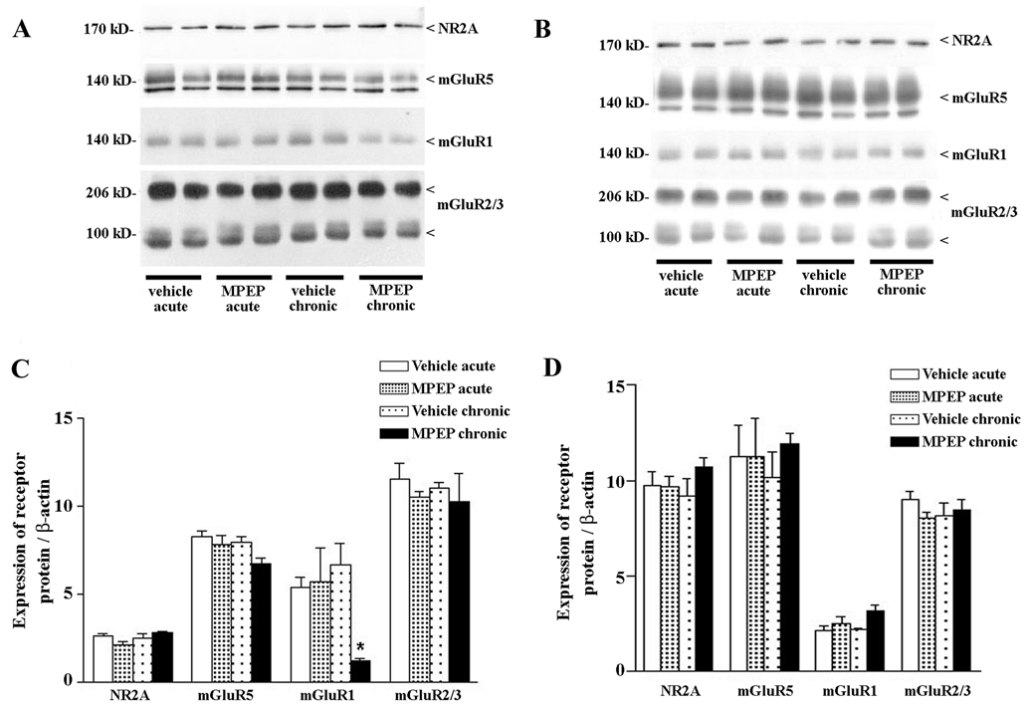
Prolonged mGluR5 antagonism results in reduced expression of mGluR1 in the dentate gyrus, but not in CA1 region. Expression of mGluR5, mGluR2/3 and NR2A remain unchanged both in the dentate gyrus and CA1. A, B. Western blot analysis of NR2A, mGluR5, mGluR1 and mGluR2/3 receptors in the dentate gyrus (A) and in the CA1 region (B). Each lane shows receptor expression in individual animals from control and treated groups. C, D. Densitometric analysis of NR2A, mGluR5, mGluR1 and mGluR2/3 expression are shown in the dentate gyrus (C) and in CA1 (D), following acute or prolonged treatment with MPEP. Each individual value was normalized by the expression of ß-actin. Values are mean±S.E.M of six individual determinations. Asterix denotes statistically significant difference (p<0.05).

Western blot analysis of NR2A protein showed a net 170 kD band (Figures 2A and 2B). mGluR5 antibodies labelled two bands (at about 140 kD), with the upper one corresponding to mGluR5 receptor monomers. We confirmed this previously by comparing the blots with those obtained by neonate brain tissue, where the mGluR5 receptor protein is known to be markedly up-regulated [24]. The mGluR1 receptor antibody labelling showed a 140 kD band corresponding to receptor monomers, whereas mGluR2/3 antibodies labelled two monomeric bands at about 100 kD, and an additional higher molecular weight band which corresponds to receptor dimers.

Following three daily injections of MPEP, expression of mGluR1a receptors was reduced in the dentate gyrus whereas expression of NR2A and mGluR2/3 receptors was unchanged in this subregion. Additionally, a trend towards a decrease in mGluR5 receptor expression was evident in the dentate gyrus (Figure 2C). No significant change in the expression of NR2A, mGluR1a mGluR5 and mGluR2/3 receptors was seen in the CA1 region compared to controls (Figure 2D). Hence, the prolonged antagonism of mGluR5 leads to a significant decrease in expression of mGluR1 only, and this effect is specific to the dentate gyrus.

### Prolonged mGluR5 antagonism results in impairment of expression of long-term potentiation in the dentate gyrus *in vivo*, which is attenuated by allosteric potentiation of mGluR1

**Figure 3:**
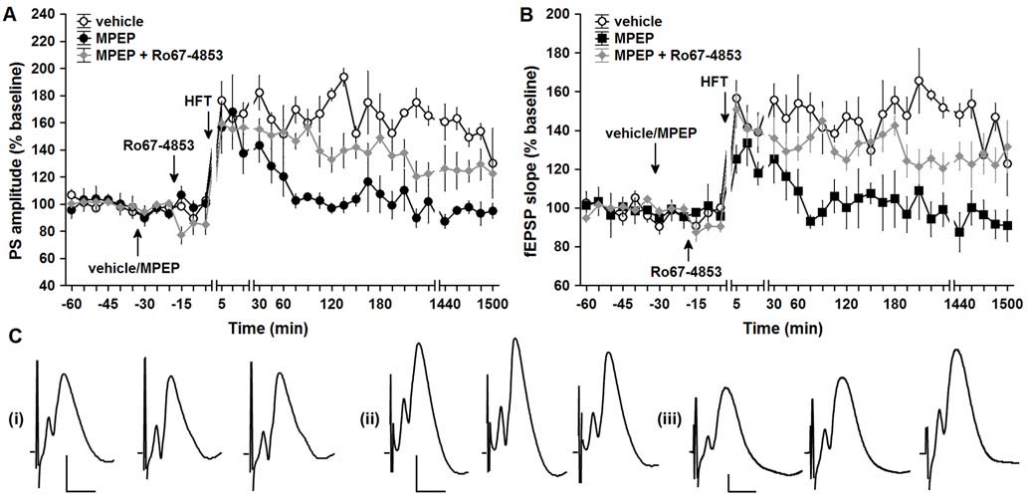
An impairment of LTP occurred after prolonged mGluR5 antagonism is partly mediated by mGluR1 in the dentate gyrus. A, B. HFT induces robust LTP of PS amplitude (A) and fEPSP slope (B) in controls treated with vehicle for three days (open circles). Prolonged application of MPEP (filled circles) results in a significant impairment of LTP induced 30 min after the final injection, compared to vehicle-injected controls. Potentiation of mGluR1 by Ro67-4853 after treatment with MPEP (grey diamonds) led to a significant recovery of late-LTP. Data are represented as mean±S.E.M. C. Analogues represent fEPSP responses during pre-HFT baseline, 5 min post-HFT and 24h post-HFT period, recorded after treatment with (i) vehicle, (ii) MPEP, and (iii) MPEP and Ro67-4853. Scale bars: vertical 2 mV, horizontal 5 ms.

Acute application of MPEP (1.8 µg, i.c.v) significantly impairs the late phases of LTP in the dentate gyrus *in vivo* [11]. Here, we examined whether changes in dentate gyrus LTP could be observed following daily treatment with MPEP for three days. Application of HFT to the medial perforant path elicited robust LTP in controls (n = 4) (Figure 3, open circles). Prolonged treatment with MPEP (n = 4) resulted in a prominent and highly significant impairment of LTP (F_1,168_ = 171.66, p<0.001 for PS; F_1,168_ = 189.64, p<0.001 for fEPSP slope) (Figure 3, filled circles).

Our finding that prolonged mGluR5 antagonism led to a subregion-specific down-regulation of mGluR1a in the dentate gyrus, suggested that the prominent impairment of LTP seen in this region could be, at least partly, associated with a decreased function of mGluR1. To clarify this possibility, we treated animals with Ro67-4853, a positive allosteric mGluR1 modulator [25]. This compound does not activate mGluR1 receptor *per se*, but potentiates the effects of mGluR1 activation by glutamate or other orthosteric agonists [25]–[27].

Ro67-4853 was administered once after the final (third) injection of either vehicle or MPEP (both n = 6), 15 min prior to HFT. Application of Ro67-4853 led to a depression of basal synaptic transmission, significant for both PS amplitude (F_1,28_ = 12.85, p<0.01) and fEPSP slope (F_1,28_ = 5.56, p<0.05), when compared with animas treated with MPEP. However, Ro67-4853 significantly reversed the impairment of LTP that was evident in animals that had undergone a 3-day treatment with MPEP only (F_1,219_ = 61.17, p<0.001 for PS; F_1,219_ = 96.13, p<0.001 for fEPSP slope) (Figures 3A and 3B, grey diamonds). Nevertheless, the magnitude of LTP in animals treated with both MPEP and Ro67-4853 was significantly lower, when compared with LTP in controls (F_1,209_ = 54.51, p<0.001 for PS; F_1,209_ = 29.49, p<0.001 for fEPSP slope). Hence, these results support our suggestion that alterations in mGluR1a expression that occur following mGluR5 antagonism, contribute to the impairments in LTP seen in the dentate gyrus following MPEP treatment.

### Prolonged mGluR5 antagonism *in vivo* enhances expression of long-term potentiation in the CA1 region *in vitro*

**Figure 4:**
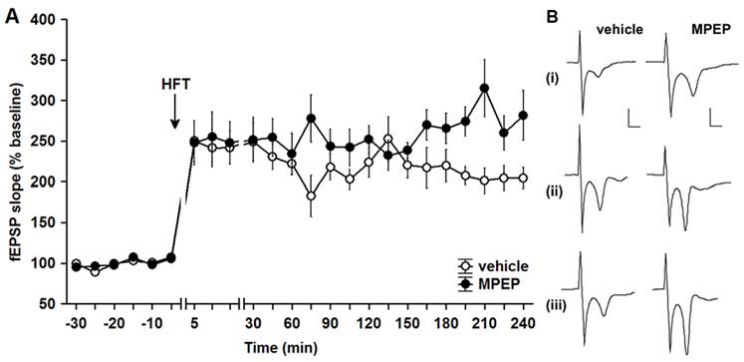
Prolonged mGluR5 antagonism *in vivo* enhances late-LTP in the CA1 region *in vitro.* A. Prolonged *in vivo* treatment with MPEP results in an enhancement of late-LTP in the CA1 region *in vitro*, when compared with controls. B. Analogues represent (i) pre-HFT, (ii) 5 min post-HFT and (ii) 4h post-HFT, at the timepoints noted, in vehicle and MPEP-treated animals. For controls: vertical bar: 2 mV, horizontal bar: 5 ms. For MPEP: vertical bar: 1 mV, horizontal bar: 5 ms. Data are represented as mean±S.E.M.

Taking into account that application of MPEP *in vivo* causes learning impairments (Figure 1), accompanied by impairment of LTP in the dentate gyrus (Figure 3), we speculated that prolonged antagonism of mGluR5 could lead to an increasing inability of hippocampal synapses to express LTP which would in turn be reflected by the exacerbation of learning performance seen in MPEP-treated rats. To exclude possible extrahippocampal influences, we chose to examine LTP expression in the CA1 region *in vitro* following 3 daily injections of either vehicle (n = 5) or MPEP (1.8 µg, i.c.v., n = 6) *in vivo*. To our complete surprise, we found that repeated application of the antagonist led to a significant *enhancement* of LTP expression *in vitro* that became apparent roughly 165 min after HFT had been applied (Figure 4A) (F_1,152_ = 23.39, p<0.001). Three-day application of MPEP did not lead to any changes in basal synaptic transmission compared to control animals (n = 5, data not shown). Bearing in mind that saturation of LTP is a mechanism by which learning can be impaired, we were interested to establish whether reduced LTP in the dentate gyrus could be related to the enhanced LTP in the CA1 region. Thus we next examined what would happen if we interfered with communication between these structures.

### Disruption of communication between the dentate gyrus and CA1 region reverses chronic effects of MPEP treatment on LTP

**Figure 5:**
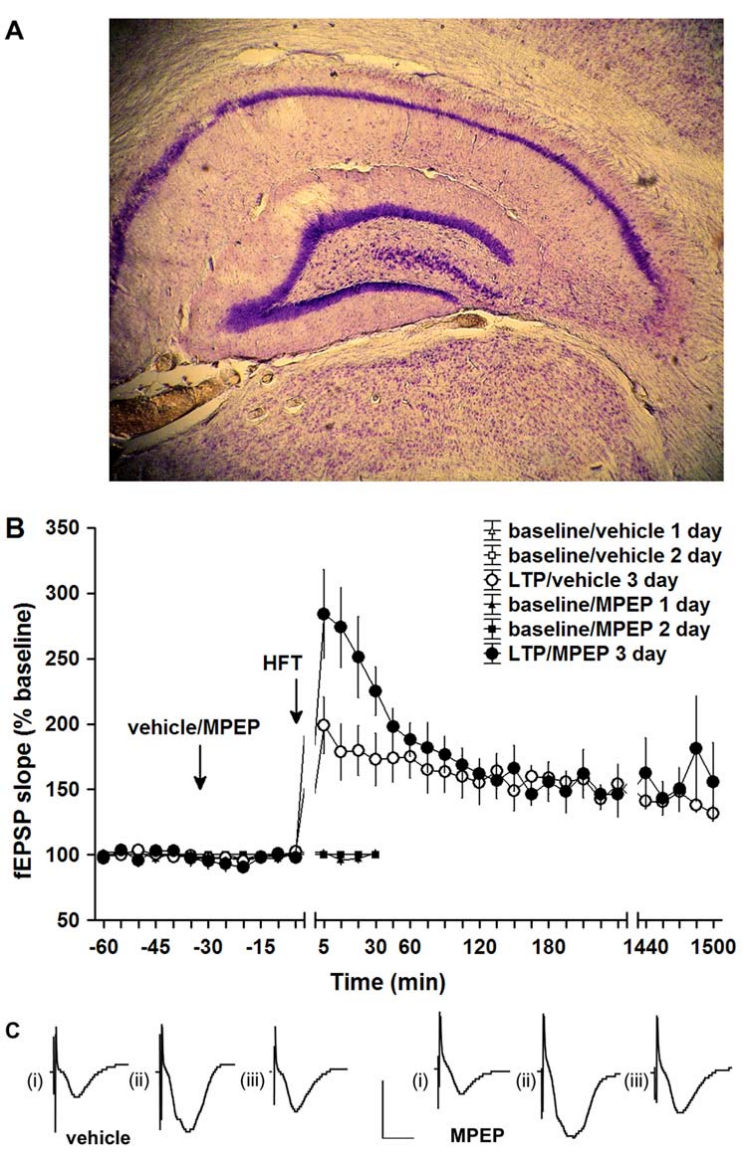
Induction phase of LTP is enhanced in CA3-lesioned animals after prolonged MPEP treatment. A. A transverse section through the rat brain at the level of ca. 3.1–3.3 mm posterior to bregma, demonstrating the lesioning of the hippocampal CA3 region as a result of kainate injection. B. Daily administration of MPEP for three days in CA3-lesioned rats resulted in an enhanced induction of LTP in CA1 region in comparison with CA3-lesioned animals that were treated with vehicle. Data are represented as mean±S.E.M. C. Analogues represent (i) pre-HFT, (ii) 5 min post-HFT and (iii) 24h post-HFT, in CA3-lesioned animals following treatment either with vehicle or MPEP. Vertical bar: 2 mV, horizontal bar: 5 msec.

To determine whether changes in synaptic processing in the dentate gyrus could have contributed to the altered LTP that we observed in the CA1 region of animals that underwent prolonged antagonism of mGluR5, we lesioned the CA3 region in another group of rats, and repeated the MPEP experiments. Application of kainate (0.5 µg in 1 µl) resulted in a pronounced but selective lesion of the CA3 region (Figure 5A). Basal synaptic transmission in CA3-lesioned animals was stable throughout the recording period following prolonged treatment either with vehicle (n = 5) or MPEP (1.8 µg, n = 4) (Figure 5B).

No difference in the late phase of CA1 LTP was found between two groups of CA3-lesioned animals that were repeatedly treated either with vehicle or with MPEP (Figure 5B). Interestingly, the induction phase of LTP was significantly enhanced after prolonged mGluR5 antagonism, when compared with vehicle-treated rats (F_1,151_ = 14.75, p<0.001). Thus, lesioning of CA3 prevented the MPEP-mediated alterations of LTP in the CA1 sub-region, consistent with the possibility that effects were driven by mechanisms originating from the dentate gyrus.

### Hippocampal network activity is altered in animals treated with MPEP

The dentate gyrus serves as an important information gateway to the hippocampus, particularly during novel spatial exploration in rats. Theta-gamma activity may underlie encoding of learned information which is relayed through the CA3 region to the CA1 region where final encoding of the memory trace may occur [28]. Considering the role of the dentate gyrus in the genesis and/or maintenance of hippocampal theta and gamma oscillations [29]–[31], we speculated that antagonism of mGluR5 could lead to disturbances in network activity in the dentate gyrus which in turn could have repercussions on the ability of the dentate gyrus to process synaptic information. We therefore examined whether repeated injection of MPEP could change the power of theta and gamma oscillations in the dentate gyrus.

#### Prolonged antagonism of mGluR5 suppresses theta (5–10 Hz) oscillations in the dentate gyrus in vivo

**Figure 6:**
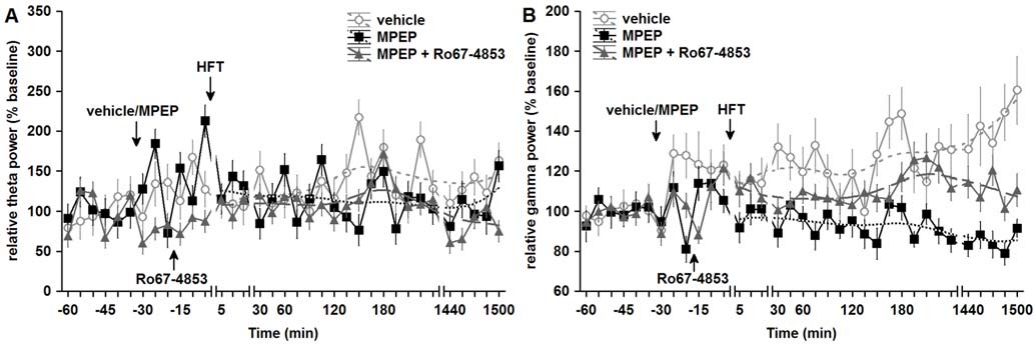
Hippocampal network activity is altered in animals treated with an mGluR5 antagonist. A, B. Relative theta (5–10 Hz, A) and gamma (30–100 Hz, B) power in the dentate gyrus prolonged treatment with vehicle (open circles), MPEP alone (filled squares) or MPEP with Ro67-4853 (filled triangles). Note that potentiation of mGluR1 with Ro67-4853 partially prevented the suppression of gamma oscillations, which was caused by prolonged MPEP treatment. The values represent averaged data for five 4-s epochs selected after test-pulses at each time-point and normalised to pre-injection values (mean±S.E.M.). Curve-fits are plotted based on distance weighted least squares for time-points after HFT.

The analysis of the period after injection, but prior to HFT revealed that the effect of injection *per se* was significant in all three groups, with higher relative theta power after injection found in rats treated with vehicle (F_1,221_ = 7.45, p<0.01) and MPEP (F_1,224_ = 12.15, p<0.001) (Figure 6A). Application of HFT did not immediately change theta power in controls, whereas in the MPEP-treated group, HFT resulted in its significant decrease (F_1,531_ = 8.88, p<0.01), when compared with respective pre-HFT periods. In animals treated with MPEP and Ro67-4853, a decrease of the relative theta power occurred after MPEP injection (F_1,254_ = 8.73, p<0.01), which might be caused by the additional handling necessary for removal of the injector from cannula after MPEP injection, and insertion of the second one with Ro67-4853 injection solution. However, application of HFT 15 min after Ro67-4853 injection led to a significant increase of the relative theta power (F_1,737_ = 6.12, p<0.05).

The analysis of the effect of prolonged mGluR5 antagonism on the relative theta power in the period after HFT revealed its significance (F_1,855_ = 9.28, p<0.01) (animals treated with both MPEP and Ro67-4853 were not included into this analysis). Generally, controls showed a higher relative theta power than MPEP-treated animals (Figure 6A). No significant effect of Ro67-4853 on the relative theta power in the period after HFT was found, reflecting a similar pattern of changes in animals treated either with MPEP only, or MPEP and Ro67-4853. Thus, these data demonstrate that in post-HFT period, MPEP suppresses the relative theta power, which remained stable in controls after HFT. This effect of MPEP is unlikely to be associated with down-regulation of mGluR1, because the allosteric potentiation of mGluR1 function by Ro67-4853 did not prevent this suppression.

#### Prolonged antagonism of mGluR5 suppresses gamma (30–100 Hz) activity in the dentate gyrus in vivo; potentiation of mGluR1 attenuates this suppression

Generally, the mean values of the relative gamma power varied over the course of experiments in a narrower range than the relative power of theta oscillations (Figures 6B). In animals repeatedly injected with vehicle, but not MPEP, a significant increase of the relative gamma power following final injection was seen (F_1,221_ = 12.53, p<0.001). Further, a striking difference was found between controls and MPEP-treated animals with regard to responses in the gamma frequency band to HFT. In controls, a trend towards HFT-triggered increase of the relative gamma power was found (p<0.11). However, in animals repeatedly injected with MPEP, application of HFT led to a highly significant *decrease* of the relative gamma power in comparison with pre-tetanisation levels (F_1,531_ = 11.94, p<0.001). No significant effect of HFT on gamma power was found in animals that received Ro67-4853. Hence, animals that received Ro67-4853 injection, after 3-day MPEP treatment, demonstrated neither a trend towards the increase seen in controls, nor a significant decrease of the relative gamma power such as that seen in rats treated only with MPEP.

The results of an overall analysis demonstrated that the effect of MPEP on the relative gamma power in post-tetanisation period was highly significant (F_1,855_ = 138.70, p<0.001), reflecting *lower* values of the relative gamma power in MPEP-treated animals compared to controls. The effect of mGluR1 potentiation by Ro67-4853 was also significant in the period after HFT (F_1,1074_ = 74.31, p<0.001), reflecting higher relative gamma power in Ro67-4853-treated group, than in rats treated with MPEP only.

In summary, we found that impairment of LTP after prolonged treatment with MPEP was accompanied by a significantly *lower* gamma power when compared with animals treated repeatedly with vehicle; effects were reversed by treatment with the mGluR1 potentiator supporting that MPEP-mediated changes in mGluR1 expression may cause this effect.

## Discussion

These data provide an intriguing insight into the role of mGluR5 in spatial learning and LTP *in vivo* and *in vitro*, and reveal the mGluR5-dependency of LTP, network activity and learning in the hippocampus. Consistent with earlier data [10]–[11], [18] and with findings using mGluR5 knockout mice [32], we show here that prolonged mGluR5 antagonism leads to a strong impairment of both reference and working memory. Our data complement the results of a recent study showing a significant improvement in Y-maze spatial alternation task after post-training allosteric potentiation of mGluR5 [33]. We have shown here that the impairment of spatial memory resulting from mGluR5 inhibition is associated with enhanced LTP in the CA1 region, and both impaired LTP and suppressed oscillatory activity in the dentate gyrus. Interestingly, a reduced expression of mGluR1a in the dentate gyrus may be the key to these alterations. These findings offer a fundamental insight as to how information processing is driven by the hippocampus: mGluR5-dependent synaptic plasticity in the dentate gyrus, which is tightly linked to theta-gamma network oscillations, drives plasticity levels in the CA1 region. This interplay might be essential for long-term information storage and formation of persistent memories. These observations are consistent with recent reports of a division of labour with regard to spatial memory encoding in the hippocampus [2], [4], [34]–[35].

### Role of mGluR5 and synaptic plasticity in spatial memory encoding

An intriguing finding of this study was that prolonged mGluR5 inhibition enhanced CA1 LTP. One could speculate that the enhanced CA1 LTP promotes a tendency of the synapses to rapidly reach saturation, which in turn impairs their ability to store new information or consolidate established memory traces. Accordingly, complete saturation of LTP through repeated electrical stimulation prevents learning in a water maze [36]–[38]. Furthermore, LTP saturation as a result of synchronous discharges may comprise the mechanism whereby learning is impaired in chronic epilepsy [39]. Thus, when mGluR5 is suppressed for a long time, spatial learning events, would lead to excessive LTP in CA1 (e.g. synaptic saturation) which impairs the ability of the synapse to accurately encode learned information.

### mGluR5 is particularly important for late-LTP

In many *in vitro* studies, where the contribution of group I mGluRs to hippocampal synaptic plasticity was cast into doubt, the effect of mGluR ligand was followed for at most 60 min after induction of LTP or LTD [40]–[44]. *In vivo*, effects of group I mGluR activation/inactivation typically become apparent between 90 and 120 min after the induction of synaptic plasticity [3], [10]–[11], [45], suggesting that mGluRs are important for the expression of the late phases of LTP. We followed LTP in hippocampal slices for 4 h after HFT. Late expression of LTP was impaired with effects becoming apparent roughly 2 h after tetanisation. Moreover, application of MPEP after the tetanus also impairs late-LTP [46].

Taken together, these data suggest that regulation of LTP by mGluR5 involves two independent aspects: regulation of the NMDAR-dependent induction phase of LTP, presumably *via* modulation of NMDA receptor currents [47]; and regulation of the protein synthesis-dependent phase of LTP [16]–[17]. Induction of LTP *in vivo* is associated with increased expression of mGluR5 [19], whereas group I mGluR-mediated slow-onset potentiation has been shown to result in increased expression of calcium binding proteins [48]. Pharmacological activation of group I mGluRs leads to dendritic protein synthesis [49], and stimulates the mitogen-activated protein kinase pathway, thereby promoting gene expression. Therefore, antagonism of mGluR5 by MPEP may lead to interference of the synthesis of mGluR5 protein and/or of other proteins necessary for LTP maintenance.

### Role of network activity in the dentate gyrus

Several lines of evidence show that information can be processed and stored not only at the synaptic level, but also within distributed networks, built of neuronal elements that show different, but coordinated patterns of activity. One of the main features of such neuronal assemblies is their oscillatory activity, which coordinates or sets the working mode of individual elements within the network and, in the same time, dynamically reflects the processes ongoing in the network. In freely moving animals, theta and gamma network activity occurs in close association, when an animal is aroused, learns new information (encoding) and/or when the memory of this information is replayed (retrieval/recall) (see for review: [29], [31], [50]–[54]). Characteristic to the hippocampal formation, theta activity appears to segregate neuronal discharges within the gamma frequency range into distinct epochs. These epochs may comprise the first stage in the formation of a memory engram [28]; [50]. Furthermore, LTP induced by HFT is believed to reflect synaptic encoding of spatial information [1]. Here, we found that prolonged MPEP treatment suppressed theta activity in post-HFT period. Although, not so straightforward as for gamma activity, this effect co-occurred with an impairment of LTP in the dentate gyrus, showing the importance of theta oscillations for expression/maintenance of LTP. This is in line with data showing a higher power of theta in the “aroused” brain state when acquisition and/or encoding of sensory information should be facilitated [29]. Additionally, it has been previously shown that i*n vitro* activation of mGluR5 can evoke theta frequency oscillations in CA3 network [21]. Furthermore, in our study, treatment with Ro67-4853, aimed to restore mGluR1-mediated signalling after treatment with MPEP, did not prevent MPEP-induced suppression of the relative theta power or had any significant effect in the period after tetanisation, suggesting that under these conditions mGluR1 is less involved in maintenance of theta oscillations in the dentate gyrus.

One of the key findings of our study is that the pronounced impairment of LTP, found in the dentate gyrus after prolonged mGluR5 antagonism, was associated with a significant HFT-induced decrease of the relative gamma power. On the other hand, normal LTP, in animals repeatedly injected with vehicle, was accompanied by a trend towards higher gamma power. These results indicate a positive relationship between the expression of LTP and the power of gamma oscillations. Confirming this, mGluR1 potentiation after prolonged exposure to MPEP not only partially rescued LTP, but also prevented HFT-induced decrease of the relative gamma power in the dentate gyrus.

If the power of gamma oscillations reflects the synchronization process in neuronal assemblies within a network [29]–[30; 50], the decrease of both gamma and theta power in the dentate gyrus might indicate that a prolonged mGluR5 blockade led to a disturbance of the precise temporal organisation of activity within the interneuronal network. This is consistent with previous reports that activation of mGluR5 in layer V pyramidal neurons increases their intrinsic excitability, which improves input-output function and increases temporal precision of the neuronal discharges [55]. On the other hand, our results indicate that the suppression of gamma activity seen was driven by the down-regulation of mGluR1 in the dentate gyrus. Transgenic animals which lack mGluR1 have been reported to have altered LTP *in vivo*, no change in (or alternatively impairment of) LTP *in vitro* and impaired learning [56]–[57]. It has been reported that mGluR1 activation leads to synchronized oscillations at alpha and theta frequencies in the lateral geniculate nucleus [58] and to enhanced slow (<1 Hz) EEG rhythm in the thalamus [59]. As is the case for mGluR1, mGluR5 activation increases neuronal synaptic excitability by increasing synchrony between cells and driving associated network activity, whereby mGluR5 is mainly responsible for *initiation* of bursting activity within hippocampal neuronal networks [60]. In contrast, mGluR1 mediates burst *duration* and *amplitude* [61]. Thus, the disruption of mGluR1a expression may comprise a key mechanism underlying the deficits in LTP in the dentate gyrus and suppression of gamma oscillations which arise following prolonged antagonism of mGluR5.

### The dentate gyrus as a regulator of plasticity

Our data suggest that the enhancement of LTP in the CA1 region may be driven by changes in synaptic efficacy mediated by mGluR5 in the dentate gyrus, as lesioning of the CA3 region prevented the enhancement of late-LTP in CA1 after prolonged treatment with MPEP. One can expect that lesion of intrahippocampal administration of kainate would produce not only cell death in CA3 area, and loss of Schaffer collaterals, but also change the firing mode of surviving neurons and generally increase their excitability [62]. Furthermore, kainic lesions may produce synchronous hyperexcitability of CA1 neurons in vitro [63]. In a recent study, a more robust electrically induced LTP in the CA3 region was reported in animals after pilocarpine-induced status epilepticus than in controls [64]. Putative increased excitability of CA1 neurons did not appear to be a notable factor in our study. In fact, we observed that prolonged mGluR5 antagonism enhanced late LTP in CA1 area in slices from intact animals, but not in CA3-lesioned rats. These data suggest that synaptic activity in the dentate gyrus, mediated by both mGluR5 and mGluR1 activation, maintains CA1 LTP in a functional dynamic range and serves to prevent its elevation to saturation levels. This agrees with reports that mGluR1 can influence GABA transmission and thereby alter LTP levels [57], [65]. The tight interplay between mGluR1 and mGluR5 activation may explain why both of these receptors appear critical for expression of hippocampal synaptic plasticity *in vivo* [11], [66].

### Overview

In conclusion, we demonstrate here, that the impairment in long-term memory elicited by *in vivo* antagonism of mGluR5 is associated with deficits in LTP in the dentate gyrus *in vivo* and an enhancement of LTP in the CA1 region *in vitro*, suggesting that mGluR5 is important for maintaining LTP within a physiological range. Deficits in this regulation may lead to saturation of LTP and inhibition of LTP-dependent spatial learning. Both the inhibition of mGluR1 expression and suppression of both theta and gamma oscillatory activity caused in the dentate gyrus by chronic mGluR5 antagonism appear to contribute importantly to the impairment of initial encoding of sensory information. These findings suggest that mGluR5 is intrinsically involved in the formation of hippocampal LTP and information processing. The mechanisms underlying this effect involve, in addition to mGluR5-mediation of intracellular calcium concentrations, regulation of mGluR1 receptor expression and the modulation of network activity in theta and gamma frequency ranges in the dentate gyrus. Most strikingly, our data support that the dentate gyrus is intrinsically involved in regulating plasticity levels within the CA1 region and that a tightly regulated interplay between these structures is essential for the encoding of spatial memory by synaptic plasticity.

## Materials and Methods

### Compounds and Drug Treatment

The allosteric mGluR5 antagonist 2-methyl-6-(phenylethynyl)pyridine (MPEP) (Biotrend, Germany) was dissolved in 0.9% NaCl. The positive allosteric mGluR1 modulator (9H-xanthene-9-carbonyl)-carbamic acid butyl ester (Ro67-4853) was synthesized as previously described [25]–[26] and was prepared as a stock solution in DMSO and then diluted with 0.9% NaCl so that the concentration of DMSO in solution for injection was <0.05%. MPEP, Ro67-4853 or vehicle were injected in a 5 µl volume over a five minute period into the lateral cerebral ventricle (l.c.v.) via a Hamilton syringe. Injections were given three times at 24h intervals with the final injection being given 30 min prior to the tetanisation (LTP experiment). Ro67-4853 was injected once 15 min prior to tetanisation. Throughout the experiments, injections were administered following measurement of the baseline for 30 minutes. In LTP experiments, a tetanus was applied 30 min following injection, with measurements then taken at t = 5, 10, 15 and then 15 min intervals up to 4 h, with additional measurements taken after 24h. In days 1 and 2 of the prolonged treatment, fEPSPs were measured for one hour after injection without any additional stimulation.

### Western Blot Analysis

Following treatment with either vehicle or MPEP, brains were removed 24h after the last of the three daily injections of vehicle or MPEP. Hippocampal subregion dissections were immediately conducted (on ice) under a microscope. The CA1 and dentate gyrus were isolated and stored separately at −80°C for subsequent biochemical analysis. Tissues were then homogenized at 4°C in 50 mM Tris-HCL buffer, pH 7.4, containing 1 mM EDTA, 1% Triton X-100, 1 mM PMSF, 1 µg/ml aprotinin, 1 µg/ml pepstatin, and 1 µg/ml leupeptin. After sonication, 3 µl of total extract were used for protein determinations. One hundred µg of protein extract were resuspended in SDS-bromophenol blue reducing buffer with 40 mM DTT. Western blot analyses were carried out using 8% SDS polyacrylamide gels which were electroblotted onto PVDF membranes (Biorad, Italy); filters were blocked for 1 h in TBS-T buffer (100 mM Tris-HCL; 0.9% NaCl, 0.1% Tween 20, pH 7.4) containing 5% non-fat dry milk. Blots were then incubated for 1h at room temperature with primary polyclonal antibodies (1 µg/ml) which recognize a specific carboxy-terminal epitopes of mGlu1a, mGluR2/3, mGluR5 and NR2A receptors (Upstate Biotechnology, Milan, Italy) or monoclonal antibody to label β-actin (Sigma, St. Louis, MO, USA) (actin blottings were used to quantify the amount of protein charged per lane in each gel run), washed with TBS-T buffer and then incubated for 1 h with secondary antibodies (peroxidase-coupled anti-rabbit or anti-mouse) (Amersham, Piscataway, NJ, USA), diluted respectively 1:10000 or 1:5000 with TBS-T. Immunoreactivity was revealed by enhanced ECL (Amersham). Optical density readings for the detected bands were determined using a computer-assisted densitometry program (ONE-Dscan 2.03, Scanalytics). Statistical analysis was performed by ANOVA followed by Fisher LSD test.

### Behavioural Analysis

#### The radial maze

The radial maze consisted of a central octagonal platform from which 8 arms radiated (total length 160 cm). The construction and implementation of the maze, as well as behavioural analysis of learning was exactly as described previously [11]. Nine- to thirteen-week old male Wistar rats which had undergone implantation of an injection cannula, were used for the behavioural study.

On training days four arms were baited with a single food pellet (“Dustless Precision Pellet”, Bioserv, Frenchtown, NJ, USA). For each animal a different constellation of baited arms was randomly chosen. This constellation remained constant throughout the training days. Thirty minutes prior to the commencement of each trial MPEP or vehicle injection was applied in a volume of 5 µl with exactly the same procedure as for electrophysiological experiments.

#### Performance scoring

Entry into an unbaited arm or entry into a baited arm without removing the food pellet was scored as a reference memory error. Reentry into a baited arm from which the food pellet had already been retrieved, or reentry into an unbaited arm was scored as a working memory error. Animal activity was determined by a simple calculation based on the amount of time spent in the maze and the number of arms crossed: 
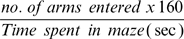
, where 160 equals the length of the maze from arm tip to opposite arm tip.

#### Behavioural data analysis

Working and reference memory error data from each of the three trial days were analysed for each individual and expressed as mean data per trial day. ANOVA was used to determine statistical significance. The probability level interpreted as statistically significant was p<0.05.

### In vivo electrophysiology

Seven-to-eight week old male Wistar rats underwent electrode implantation into the dentate gyrus or CA1 region as described previously [3], [67]. For recordings from the CA1 region, the recording electrode was placed 2.8 mm posterior to bregma and 1.8 mm lateral to the midline. The stimulating electrode was inserted 3.1 mm posterior to bregma and 3.1 mm lateral to the midline (coordinates based on: [68]). For recordings from the dentate gyrus, the recording electrode was placed 3.1 mm posterior to bregma and 1.9 mm lateral to the midline, whereas the stimulating electrode was inserted 6.9 mm posterior to bregma and 4.1 mm lateral to the midline. To enable i.c.v. drug injection, animals underwent implantation of a cannula in the lateral cerebral ventricle (0.5 mm posterior to bregma; 1.6 mm lateral to the midline). The animals were allowed between 7–10 days to recover from surgery before experiments were commenced. Experiments were carried out using 9–13 week old freely moving rats, and consistently conducted at the same time of day (commencing 9.00am). Baseline experiments to confirm stability of evoked responses were routinely carried out (at least 24h) before electrophysiological experiments were conducted.

The present study was carried out in accordance with the European Communities Council Directive of November 24^th^, 1986 (86/609/EEC) for care of laboratory animals and after approval of the local ethic committees (senate of Berlin or Bezirksamt Arnsberg).

### Kainate Lesions

In animals that underwent bilateral lesions of the CA3 region with kainic acid (Biotrend, Germany), additional drill holes (3.5 mm posterior to bregma, 3.2 mm lateral to the midline) were made, on each side of the midline, during electrode implantations. Prior to electrode implantation, a cannula, attached via polyethylene tubing to a Hamilton syringe, was lowered into the CA3 region (depth 3.0–3.3 mm) and kainic acid (0.5 µg dissolved in a 1 µl injection volume of 0.9% NaCl) was injected over a 10 min period. Thirty minutes later, the cannula was removed, the drill hole was sealed with cyanoacrylate glue and dental cement, and the injection was repeated in the opposite hemisphere in the same way. In this case however, the drill-hole was not sealed after injection to enable subsequent implantation of the electrodes. Procedures were then followed as described above. Following conclusion of the experiments, post mortem histological analysis was conducted to ensure that accurate lesioning of the CA3 region had occurred. Animals that expressed spontaneous epileptic seizures after recovery period were excluded from the study.

### Measurement of Evoked Potentials

Responses were evoked by stimulating at low (test-pulse) frequency (0.025 Hz, 0.2 ms stimulus duration, 16 kHz sampling rate) as described previously [3], [68]. In the dentate gyrus, LTP was induced by a HFT of 200 Hz (10 bursts of 15 stimuli, 0.2 ms stimulus duration, 10 s interburst interval), using a stimulus amplitude which was the same as that used for recordings. In the CA1 region, LTP was induced by a HFT of 100 Hz (10 bursts of 10 stimuli, 0.1 ms stimulus duration, 10 s interburst interval) and a stimulus amplitude that comprised 20% of the maximum determined from the input-output analysis.

### *In Vivo* Treatment Prior To *In Vitro* Experiments

Under anaesthesia, a cannula was implanted into the lateral cerebral ventricle of seven- to eight-week old male rats. After 7–10 days recovery from surgery, MPEP was administered (1.8 µg in 5 µl) three times at 24h intervals (procedures as described above). Further 24h later hippocampi were dissected for *in vitro* electrophysiological analysis.

### *In Vitro* Experiments

Seven- to eight-week old male Wistar rats were anesthetised with ether and then decapitated. Brains were dissected in ice-cold artificial cerebrospinal fluid. Immediately after preparation, slices (400 µm) were placed on a nylon net in a 2 ml circulation chamber at the interface between incubation medium and a humidified atmosphere of 95%O_2_/5%CO_2_ and continuously perfused (with a constant flow rate of 3 ml/min) with an oxygenated Ringer's solution (in mM: NaCl 124, KCl 4.9, KH_2_PO_4_ 1.2, MgSO_4_ 1.3, CaCl_2_ 2.5, NaHCO_3_ 25.6, D-Glucose 10) at 35°C. Following 30 min equilibration, the slices were submerged by filling the chamber to a volume of 3 ml with warmed (35°C) O_2_/CO_2_ Ringer's solution. The flow rate was then adjusted to 0.8 ml/min. Monopolar platinum-tipped silver chloride electrodes were positioned in the *stratum radiatum* of the CA1 region for stimulation and in the CA1 dendritic area for recording. Two stimulation electrodes were placed on either side of the recording electrode at adequate distance to stimulate separate inputs [69]–[70]. Measurement of evoked potentials was conducted as described below for the *in vivo* recordings. LTP was induced in one stimulation input only, whereas the other input was used to generate test-pulse responses. HFT consisted of 3 stimulus trains of 100 pulses at 5 min intervals.

### Analysis of Network Activity

Intrahippocampal electroencephalogram (EEG) was recorded from the dentate gyrus granule cell layer. EEG was sampled at 0.5 or 1 kHz and stored on hard disc for further off-line analysis. To evaluate spectral power of theta (5–10 Hz) and gamma (30–100 Hz) activity, 4-s long epochs, 1 s after each test-pulse, were selected. Fourier analysis (Hamming window function, 1024 or 2048 frequency bins for 0.5 or 1 kHz records, respectively) was performed in artefact-free epochs using “Spike2” software (Cambridge Electronic Design, Cambridge, UK). The absolute values of spectral power for each individual animal were normalized to baseline (with mean values during baseline pre-injection period taken as 100%) and the relative values were used further for statistics. Generally, the results of Fourier analysis of five epochs were averaged for each time-point. The statistical treatment and analysis of data included the analysis of variance and *post hoc* Fisher LSD test. By means of ANOVA the effects of injection itself and of HFT on spectral power were estimated. Two-way ANOVA was used to evaluate the effects of time and of MPEP treatment, as well as of their interaction. In order to separate drug effect from other effects, two-way ANOVA was performed separately for periods of baseline measurement, after injection but pre- HFT, and post-HFT.

### Analysis of Electrophysiological Data

In all electrophysiological experiments, data were expressed as mean % pre-injection values ± standard error of the mean. The significance of factorial effects and differences between samples was estimated by means of ANOVA/MANOVA and *post hoc* Student's t- and LSD-tests in STATISTICA data analysis software system (StatSoft, Inc., Tulsa, OK, USA). The probability level interpreted as statistically significant was p<0.05 (*).
