# Correction: MGluR5 Mediates the Interaction between Late-LTP, Network Activity, and Learning

**DOI:** 10.1371/annotation/58313075-ff2e-4268-9272-e942aed8d2f6

**Published:** 2008-05-28

**Authors:** Arthur Bikbaev, Sergey Neyman, Richard Teke Ngomba, P. Jeffrey Conn, Ferdinando Nicoletti, Denise Manahan-Vaughan

The fourth author's name appears incorrectly in the author list and in the citation. It should be P. Jeffrey Conn in the author list and Conn PJ in the citation.

